# From translation to stabilization and degradation: A multifaceted approach for the treatment of superoxide dismutase 1–associated amyotrophic lateral sclerosis

**DOI:** 10.4103/NRR.NRR-D-25-00778

**Published:** 2025-11-25

**Authors:** Christen G. Chisholm, Luke McAlary, Jeremy S. Lum

**Affiliations:** Molecular Horizons and School of Science, University of Wollongong, Wollongong, NSW, Australia; Molecular Horizons and School of Medical, Indigenous and Health Sciences, University of Wollongong, Wollongong, NSW, Australia

Superoxide dismutase 1 (SOD1) is a thermodynamically stable, zinc and copper binding homodimeric enzyme responsible for breaking down superoxide radicals. More than 200, mostly missense, mutations spread throughout the *SOD1* gene are associated with the fatal neurodegenerative disease, amyotrophic lateral sclerosis (ALS). A unifying feature of ALS-associated SOD1 mutations is the destabilization of the SOD1 protein structure, increasing the propensity for misfolding and subsequent pathological aggregation. Post-mortem analysis of SOD1-associated ALS tissue shows the accumulation of misfolded SOD1 protein and ubiquitinated SOD1 inclusions within motor neurons. Misfolded SOD1 accumulation and aggregates are implicated in cellular dysfunction via a number of disparate but critical processes, including endoplasmic reticulum stress, oxidative damage, proteasome dysfunction, axonal transport abnormalities and synaptic dysfunction; culminating in motor neuron degeneration associated with ALS. As a result, misfolded and aggregated SOD1 is a primary target for therapeutic investigation in SOD1-associated ALS. Some of these approaches have shown preclinical and clinical promise, but often these therapies are targeted against a single component of disease. The cancer field has made great clinical strides utilizing a multi-pronged strategy to treat various forms of cancer. Patients are treated with a combination of multiple chemotherapy agents, radiation, surgery and/or immunotherapy to produce more effective therapeutic outcomes. In a similar manner, we propose that utilizing a multifaceted approach to target SOD1 across its pathogenic landscape may provide a highly feasible and more effective treatment approach.

**SOD1 antisense oligonucleotides:** The most significant advancement in the treatment of SOD1-associated ALS has been the reduction of SOD1 expression using antisense oligonucleotides (ASOs). ASOs are short, synthetic strands of nucleic acids designed to complementarily bind target mRNA, a key example being the SOD1 ASO, Tofersen (alternatively known as BIIB067 or Qalsody). Once bound, the RNA-DNA hybrid is recognized by RNase H and subsequently degraded, thus preventing translation.

Phase 1–2 clinical trials of Toferson showed a favorable safety profile and target engagement. The subsequent 28-week Phase 3 clinical trial failed to meet the primary outcome for efficacy based on the ALS Functional Rating Scale-Revised; however, a reduction in SOD1 levels in cerebrospinal fluid, combined with a reduction in plasma neurofilament light chain levels, was observed, prompting a further non-randomized open-label extension study. Following the Phase 3 trial, Tofersen was assessed in a non-randomized open-label extension study. Encouragingly, after 52 weeks of treatment, patients showed a slower decline in respiratory function and muscle strength (Miller et al., 2022). Based on these findings, Tofersen was approved by the US Food and Drug Administration for clinical use. However, the delay between biomarker response and the emergence of clinical benefit, observed only after several months, underscores the temporal disconnect between changes to SOD1 levels and clinical presentation. Due to this, a new phase 3 trial of this SOD1 ASO is currently underway to evaluate the effectiveness when initiated in pre-symptomatic SOD1-ALS carriers.

Whilst still in its infancy for clinical application, there is cautious optimism that Tofersen represents the first potential disease-modifying therapy for SOD1-associated ALS with some caveats. Firstly, it will be critical to assess whether the long-term knockdown of SOD1 is harmful. Secondly, Tofersen only decreases SOD1 levels in CSF by 20%–40% (Miller et al., 2022), permitting a large pool of *SOD1* mRNA to escape ASO-mediated degradation. The leakage of mutant *SOD1* mRNA would continue to contribute to the accumulation of misfolded SOD1. Recently, several promising studies have utilized siRNA to increase the knockdown of SOD1; however, they face their own clinical translation challenges and are unlikely to completely eliminate mutant SOD1 production, providing the need for this misfolded SOD1 to be addressed via other means.

**SOD1 pharmacological chaperones and small molecule stabilizers:** A common characteristic of ALS-associated SOD1 mutations is the propensity to destabilize the protein, causing the accumulation of toxic misfolded and aggregated species. Whilst only a small 16 kDa protein, SOD1 undergoes a series of post-translational modifications as it folds into its stable native dimeric form. Initially, newly synthesized SOD1 adopts a partially folded conformation that allows for the spontaneous binding of Zn²⁺. Subsequently, the copper chaperone for SOD1 delivers Cu²⁺ to the protein, promoting oxidation of the intramolecular disulfide bond and facilitating homodimerization of the mature SOD1 monomers, forming a remarkably stable dimer that is resistant to thermal unfolding up to temperatures of 90 °C. ALS-associated mutations alter physicochemical properties of SOD1 to varying extents, driving mutant SOD1 toward misfolding pathways at different stages of maturation and leading to mutation-specific levels of toxicity. This has prompted the notion that small molecules capable of promoting proper folding, enhancing structural stability, and preventing misfolding could serve as a promising therapeutic strategy for SOD1-linked ALS.

The numerous post-translational modifications SOD1 must undergo to reach the stable dimeric form represent sites at which SOD1 can also divert to off-folding pathways. These numerous steps in the SOD1 (mis-)folding pathway represent multiple points at which to correct and stabilize SOD1. Small molecules aimed at correctly folding and stabilizing SOD1 have shown promise in models of SOD1-ALS. We have previously provided an overview of small molecule strategies to aid SOD1 folding and blocking aggregation (McAlary and Yerbury, 2019). Experimentally, we have focused on the small molecules, CuATSM, ebselen, and telbivudine, which have been tested in humans. CuATSM increases levels of copper-bound mutant SOD1 (Hilton et al., 2017), thereby improving SOD1 stability, and is currently in Phase 2/3 clinical trials for sporadic ALS. Ebselen has been found to improve the disulphide formation and maturation of mutant SOD1 in cells and animals (Capper et al., 2018; Amporndanai et al., 2020). Whilst telbivudine is purported to bind and block interactions of the W32 residue on SOD1, an important site that governs aberrant interactions, promoting protein misfolding, aggregation, and toxicity of SOD1 (DuVal et al., 2019).

CuATSM, ebselen, and telbivudine have shown independent efficacy in SOD1 models. Recently, in an effort to take a multi-pronged approach to SOD1 misfolding, we applied a small molecule polytherapy approach. Utilizing these three small molecules in combination, we assessed its therapeutic efficacy in a SOD1-associated cell model. We observed that a CuATSM, ebselen, and telbivudine polytherapy approach was more effective in reducing mutant SOD1 aggregate formation and increasing cell viability than any single small molecule alone (Lum et al., 2025). Furthermore, polytherapy was able to provide similar therapeutic effects at lower doses than higher doses of any single drug treatment, something that may be particularly important given evidence that CuATSM may not be tolerable at high doses. Encouragingly, administration of polytherapy improved SOD1 maturation and reduced accumulation of misfolded SOD1 and motor neuron loss in the spinal cord of SOD1^G93A^ mice. Importantly, administration of our small molecule polytherapy in SOD1^G93A^ slowed disease progression and improved survival. Whilst this study only focused on three small molecules, there are several other small molecules capable of aiding SOD1 maturation that may provide added benefit. However, this proof-of-concept study demonstrates that utilizing a combination of small molecules capable of aiding correct folding or stabilizing mutant SOD1 can provide therapeutic benefits.

**Targeting SOD1 for degradation or removal:** ASO delivery and pharmacological chaperone approaches aim to combat prospective toxic SOD1 accumulation; however, evidence from patient biopsies and animal models demonstrates that misfolded and aggregated SOD1 is present at the time of diagnosis and initiation of treatment. Furthermore, toxic SOD1 species have shown prion-like propagation, seeding, and spreading along neuroanatomical tracts. Therefore, to restore cellular function and stop the prion-like spread, there is a requirement to focus on removing the existing accumulated toxic SOD1 species.

Passive immunization strategies were first explored a decade ago with the intracerebroventricular infusion of misfolded SOD1-targeting monoclonal antibodies in SOD1^G93A^ transgenic mice. This approach successfully reduced misfolded SOD1 levels, extended survival, and demonstrated the therapeutic potential of antibody-based intervention (Gros-Louis et al., 2010). More recently, a human-derived antibody to misfolded SOD1 has reached Phase 2 clinical trial after promising results preclinically. Similarly, active immunization using non-metalated forms of SOD1, either apo forms of G93A or wild-type SOD1, resulted in a delay in disease onset and extended lifespan of SOD1^G93A^ mice (Liu et al., 2012). While encouraging, the use of immunotherapy as a strategy for treating ALS has a significant drawback. It is thought to primarily target extracellular misfolded protein, whereas an intracellular targeted approach is needed to combat the toxic accumulation of cytoplasmic misfolded SOD1. Furthermore, the mechanism by which antibody therapy degrades and removes misfolded SOD1 remains unknown and may provide scope for improvement.

Targeted protein degradation is an emerging therapeutic strategy gaining traction in the cancer field. By leveraging endogenous protein quality control pathways, disease relevant proteins can be targeted for elimination. Proteolysis, autophagy, or lysosomal targeting chimeras (PROTACs, AUTACs and LYTACs) are heterobifunctional molecules consisting of two ligands joined by a flexible linker. In the case of PROTACs, one ligand binds the protein of interest (POI) while the other recruits an E3 ligase, bringing these two molecules together in proximity and facilitating the ubiquitylation and proteasomal degradation of the target protein. Once the POI is released to the proteasome, the PROTAC is recycled to target another protein, thus making the concentration of PROTAC required for effect substantially less than the one-to-one stoichiometric ratio of standard protein inhibitors. In AUTACs and LYTACs, the second ligand binds an autophagy or lysosomal receptor, tethering the POI to an autophagosome for degradation.

Traditionally, these and other targeted protein degradation modalities such as molecular glues were small molecules; however, to function effectively, the POI requires a binding pocket. Furthermore, the repertoire of E3 ligases is limited. To circumvent these restrictions, targeted protein degradation approaches have evolved to include “BioPROTACs”; genetic strategies employing naturally existing protein binding partners or antibodies to the POI and engineered E3 ligases in which the substrate recognition region had been removed. We recently screened various misfolded SOD1-specific intrabodies in pursuit of a BioPROTAC candidate capable of targeting and degrading multiple pathogenic SOD1 variants, effectively reducing aggregation in cultured cells. Utilizing CRISPR/Cas9 technology, we generated a transgenic mouse line expressing this misfolded SOD1-BioPROTAC and demonstrated that its presence significantly delays disease progression in the SOD1^G93A^ mouse model of ALS. This therapeutic effect was also associated with preservation of motor neurons, reduced accumulation of insoluble SOD1, and maintenance of neuromuscular junction integrity (Chisholm et al., 2025). Collectively, these findings offer proof-of-concept for the therapeutic potential of BioPROTACs in selectively degrading neurotoxic misfolded SOD1.

**Figure 1:**
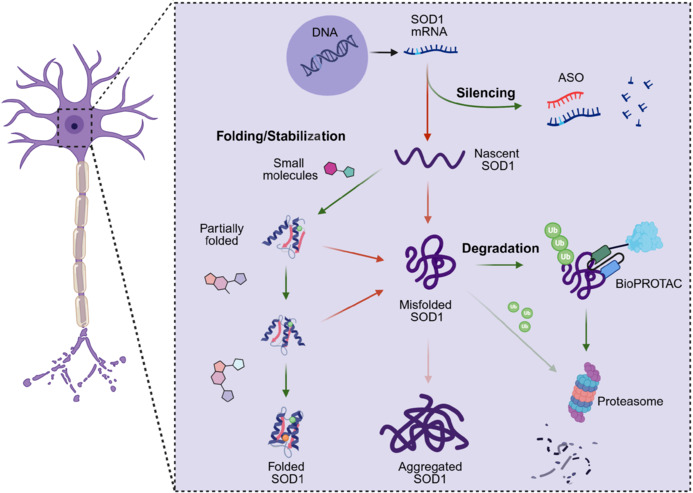
A polytherapy strategy to reduce the accumulation of toxic misfolded and aggregated superoxide dismutase 1 (SOD1). The production of toxic SOD1 species is a multiplexed process and can be targeted in a multifaceted manner. This schematic diagram illustrates several therapeutic approaches that could possibly be used in combination to reduce the toxic SOD1 load on motor neurons. These approaches include translational silencing with the use of antisense oligonucleotides (ASO), small molecules to promote maturation and misfolded specific SOD1 degraders with the use of BioPROTACs. Utilizing these approaches in combination could potentially lead to more robust therapeutic outcomes for SOD1-associated amyotrophic lateral sclerosis. Created with BioRender.com.

**Conclusions and future directions:** Since *SOD1* gene mutations were first discovered to be associated with ALS over three decades ago, there have been a myriad of approaches aiming to provide therapeutic relief. In recent years, significant clinical progress for the treatment of SOD1-associated ALS has been made in the form of Tofersen. However, this is only the first step in the personalized treatment of SOD1-associated ALS, with room for advancements. The thoroughfare of SOD1 mutation to aggregation pathology is complex and layered. Here, we have largely focused on SOD1-targeting therapies tested in our laboratory to reduce toxic SOD1 load. However, with many therapeutic avenues targeting different aspects across the SOD1 pathogenic landscape showing preclinical or clinical promise, a favorable approach will be to assess if targeting mutant SOD1 translation, folding and degradation in combination (**Figure 1**) is able to produce more robust therapeutic outcomes, similar to our strategy utilizing small molecules.

*The authors would like to acknowledge their mentor and friend, Professor Justin Yerbury. This work was the vision of Professor Yerbury, who sadly passed away to ALS, but not before inspiring, teaching, and nurturing a team of aspiring scientists to continue the fight against this devastating disease.*

*JSL obtained support from Motor Neuron Disease Research Australia in the form of a Bill Gole Postdoctoral Fellowship (PDF2307). This work was financially supported by FightMND in the form of Drug Development Grants (DDG-159 and DDG137 to JSL).*
